# Caring for Young Adults With Diabetes in the Adult Care Setting: Summary of a Multidisciplinary Roundtable

**DOI:** 10.3389/fcdhc.2022.830183

**Published:** 2022-03-22

**Authors:** Elena Toschi, Jennifer Leblanc, Samar Hafida, Sanjeev Mehta, Marilyn Ritholz, Robert Gabbay, Lori Laffel

**Affiliations:** ^1^Joslin Diabetes Center, Harvard Medical School, Boston, MA, United States; ^2^Section on Clinical, Behavioral, and Outcomes Research, Joslin Diabetes Center, Harvard Medical School, Boston, MA, United States; ^3^ American Diabetes Association, Alexandria, VA, United States

**Keywords:** young adults, adult care, transition, mental health, physical health, quality of life

## Abstract

**Aims:**

A multidisciplinary team of clinicians and researchers, patients and family members, and representative from national advocacy groups and research organization met to review the literature, highlight gaps, and identify best practices to improve adult care delivery for young adults (YA) with diabetes.

**Methods:**

The participants prepared presentations in advance, rotated through sessions, and contributed to group discussions in three areas: physical health, mental health, and quality of life (QoL). Session moderators and scribes used thematic analysis to summarize discussions for each topic.

**Results:**

Thematic analysis revealed four foci for addressing physical health, mental health and QoL: 1) best practices to facilitate the process of transfer; 2) age-specific curricula and guidelines for prevention and management of comorbidities and complications; 3) collaboration with behavioral health clinicians to address diabetes distress and mental health disorders; and 4) research on the impact of diabetes on QoL in YA.

**Conclusion:**

There was substantial interest and need among adult clinicians to work in concert with pediatric and mental health professionals to identify best practices and future directions to improve healthcare process and diabetes-related outcome measures in YA with diabetes.

## Introduction

Young adults (YA) with diabetes often experience deterioration of glycemic control, a rise in hospitalizations for acute complications, and frequent loss of follow-up (1, 2). Young adulthood is a dynamic developmental stage of life where changes occur personally, socially, and emotionally. This developmental stage also entails increasing independence from parents, change in financial status, and potential onset of psychosocial issues and risk-taking behaviors (3). During this time, healthcare is often fragmented, especially upon transfer from pediatric to adult care settings (4). Importantly, clinical trials focusing on the transition process from pediatric to adult care in YA with diabetes have yet to identify a consistently successful pathway (5–7).

The current guidelines on the transition process from pediatric to adult care systems are limited and rely heavily on expert opinion (8). Moreover, many adult clinicians lack the developmental training to engage effectively with YA in need of support in their diabetes management. Further, an increased prevalence of behavioral challenges and psychiatric disorders that may interfere with diabetes self-care have been described in YA (9–11). Lastly, quality of life (QoL) is often poor in YA with diabetes (12). A multidisciplinary team of pediatric and adult-focused clinicians, researchers, persons with diabetes, family members, and representatives from national advocacy groups convened over a 2-day meeting to review the literature, highlight gaps, discuss best practices, and identify needs for future research to improve care delivery and outcomes for YA in adult care settings. This report is the summary of the salient findings and recommendations from this roundtable conference.

## Methods

On December 5-7, 2019, the Joslin Diabetes Center organized and hosted a roundtable conference, gathering over 50 interdisciplinary thought leaders from the US and Canada to focus on delivering optimal care for YA with diabetes. The team included: pediatric endocrinologists (n=8), adult endocrinologists (n=20), family medicine physicians (n=2), nurse practitioners (n=2), nurses (n=2), certified diabetes educators (n=4), and behavioral health specialists (n=6). Many were also clinical researchers with a focus on the transition period and care of YA in adult care. Persons with diabetes, family members, and representatives from national advocacy groups and funding organizations (JDRF, ADA, Helmsley Charitable Trust, NIH, CDN, NIDDK, and the Endocrine Society) also participated in the roundtable discussions.

Three moderators were assigned to lead discussions focused on one of three sessions: 1) promoting physical health (SH), 2) optimizing mental health (MDR), and 3) preserving quality of life (SM). All participants were divided into three cohorts and rotated through each of the three topic areas. In addition, three other facilitators (LML, JL, ET) accompanied a single cohort through all three topic areas. Each session lasted two hours. Participants were asked in advance to prepare a 10-minute power-point presentation based on their experience, research, clinical approaches, and/or literature review related to Assessment, Engagement, or Intervention of one topic (physical health, mental health, or quality of life).

Following each presentation, there was a 30-minute group discussion led by the moderator and accompanying facilitator. More than 80% of the clinical participants presented a topic during the sessions. Participants contributed to group discussions in all three areas. Scribes were present in each group to take notes.

Following the day of group sessions, the session moderator, scribe, and facilitator met to discuss session findings based on the content of the presentations and the scribes’ notes to identify common themes by consensus. The next day, session moderators presented the themes to all participants for further discussion and derived consensus on each topic.

## Results

We present a summary of themes identified from the presentations and discussions that occurred over the 2-day meeting of interdisciplinary participants. The 3 topics included: 1) promoting physical health, 2) optimizing mental health, and 3) preserving quality of life. In addition, the process of transfer of care from pediatric to adult care settings became a fundamental topic for discussion during the 2-day meeting and is presented first.

The process of transfer of care, defined as “the planned, purposeful movement of young adults from child-centered to adult-oriented healthcare systems” (4), occurs during a time of change and maturation. Currently, this process remains challenging and the few randomized control studies focusing on this process have yielded little improvement in appointment attendance and glycemic control (5–7).

The participants identified three major gaps in the process of transfer: 1) a defined, straightforward, uniform process to facilitate communication between pediatric and adult clinicians, 2) clinical tools to assess maturation and readiness of YA; and 3) age-specific educational materials to help training adult clinicians in their efforts to establish a trusting relationship with YA in diabetes care delivery.

Several attendees shared their experiences in overcoming the challenge of transfer. They reported ongoing collaborations with their colleagues on the “other side”, either pediatric or adult clinicians, in efforts to establish and improve communication within and between institutions. However, these were local and isolated efforts, which relied on single providers, were time-consuming, and not yet formalized. These efforts did not address the needs of many YA who relocate geographically for school or work. The YA and parents participating in the roundtable reported that the major challenge of transfer was moving care from a familiar team to a new, unknown team, with potentially different goals and expectations.

Participants envisioned the creation of a communication system between pediatric and adult providers at different institutions, ideally incorporating a shared nationwide electronic medical record system to help communication between institutions and to track visit attendance. This effort may require several years of development and funding, while a more standardized clinical note to share – yielding a “warm handoff” – between pediatric and adult clinicians could help welcome YA into the adult care environment.

Next, participants discussed assessment of maturation. During adolescence and young adulthood, development of executive function – the ability to take on complicated tasks like diabetes self-care – occurs; this is a dynamic process that happens differently among individuals (13). The lack of or delayed maturation of executive function may lead to poor glycemic control, loss of follow-up care, low clinic attendance rates, and participation in high risk behaviors (14). Currently, there are few clinical tools for assessing maturation and readiness for self-care (15) that have been empirically validated to guide clinicians in identifying patients ready to transfer and engage in their own diabetes care. The participants recommended the development of tools for evaluation of cognitive and emotional readiness for independent self-care.

Moreover, it was recognized that adult care systems and adult clinicians are not prepared to work with YA and their family members and address the specific socioeconomic challenges of this age group (16). Participants proposed the development of a specific competency curriculum for providers who work with the YA. This curriculum would focus on helping adult providers better understand the psychosocial challenges and barriers YA face and provide guidance to adult clinicians regarding establishing trusting, interactive relationships with YA. Clinicians should also be taught to modulate their expectations to better fit the realities of this developmental stage and recognize the physical, social, and emotional burdens YA face while collaboratively identifying strategies to help improve physical and psychosocial outcomes.

### Physical Health

The participants recognized the need to develop age-specific guidelines for screening, prevention, and treatment of comorbidities and complications specific to the YA needs. They identified two major issues related to physical health: 1) the deterioration of glycemic control and increased risk of acute complications and 2) age-specific assessment, prevention, and management of comorbidities and long-term complications.

During late adolescence, glycemic control can deteriorate and often remains sub-optimal during the early years of young adulthood (17, 18). Sub-optimal glycemic control is often the result of several factors, including competing social and economic (19) demands and the ongoing maturation process, among others (20).

Among potential tools to improve glycemic control, the use of diabetes technologies – insulin pumps and Continuous Glucose Monitoring (CGM) — were identified. Many participants reported the use of CGM as best practice in this age group, especially given recent evidence of the benefits of CGM use in YA (21–23).

Participants also recommended the use of communication technologies which are commonly used by YA; i.e. text messaging, video calling, social networking, etc. (24). They sought to increase use of telehealth and text messaging to support and reinforce positive self-care behavior in between face-to-face visits and potentially to develop a web-based platform to increase peer-to-peer support. Further research in this area is needed.

Screening, assessment, and management of long-term micro- and macro-vascular complications were discussed at length for both T1D and T2D, especially given the focus on this area in adult diabetes care and the limited, if not absent, attention by pediatric providers (25, 26). Participants recognized the need and challenge of maintaining annual eye exams and kidney function testing in YA.

Cardiovascular disease (CVD) prevention was discussed. Several modifiable risk factors, like overweight/obesity and elevated blood pressure or lipids (27, 28), may already be present in YA, along with non-modifiable factors, like age of diabetes onset (29) and family history. The current clinical guidelines for prevention of CVD were developed from data in adults with T2D, and limited attention is given to YA (30). A study in adolescents on the use of statins and ACE inhibitors for early prevention of CVD did not yield benefit (31). Thus far, there is no clear indication of when and how to start prevention of CVD in YA. Moreover, statins and ACE inhibitors are contraindicated in pregnancy, making them challenging to use in women of childbearing age.

The need for further investigation in this area to elucidate how to best prevent micro and macro-vascular complications in this age group was noted (**Table 1**)

**Table 1:** Evidence, best practices, and future direction to improve the care of Young Adults with diabetes in adult care setting.

The process of transfer from pediatric to adult healthcare systems		
Evidence	Best Practices	Future Directions
Communication between pediatric and adult care is fragmented	“Warm hand-off” communicated between pediatric and adult care	Improve communication between pediatric and adult care – i.e. online registry covering all US states
Current RCT on transfer process have not been effective in improving A1c and number of follow-ups	Use of YA coordinator to facilitate transfer and improve clinical attendance	Develop tools to assess diabetes knowledge, engagement and progress over time to improve and engage YA in diabetes self-care and clinical attendance
YA have unique psychosocial needs related to their stage of life with several changes in relationship, housing, financial status etc.	Assessment of diabetes education and developmental stage to create a diabetes management plan tailored to the YA Referral to BH when available	Develop tools to assess: Maturation and readiness for transition and engagement in diabetes self-care Psychosocial needs Collaborate with behavioral health to support YA during transfer Create age-specific provider competency curriculum to establish trusty relationship and promote engagement
**Physical Health in in YA with Type 1 diabetes**		
**Evidence**	**Best Practices**	**Future Directions**
***Suboptimal Glycemic Control***		
A1c worsens during YA period A1c before transition predicts A1c after High A1c associates with low Health-Related QoL Socioeconomic disparities impact physical health Use of diabetes technology may improve glycemic control Risk of DKA and SH is higher in YA with poor glycemic control	Maintenance of clinical attendance with use of program coordinator Assessment of barriers to engage in diabetes self-care Assessment of use of diabetes technologies Assessment of social determinants of health Use of support to establish relationships with specific, measurable, achievable, realistic and time-sensitive goals (*SMART*) Discussion of mental health, sex life and alcohol/illicit drug use up front	Develop strategies to prevent deterioration of glycemic control and/or prevention of acute complication as severe hypoglycemia and DKA Validate tools to assess knowledge, readiness and barrier to diabetes management Increase use, access and engagement with diabetes technology to improve glycemic control Facilitate and increase access to telehealth/text-messaging Create peer-to-peer support systems Develop new learning and engagement software Reduce racial and socioeconomic gaps in delivery of care among YA
***Comorbidities/Complications*** High risk for CVD in T1D Early onset of T1D is associated with higher CVD risk	Screenings for complications and comorbidities Annual eye exams Assessment of whether use of ACE-I/ARB and statins is appropriate, primarily based on adult data	Develop studies to help stratify YA patients for micro and macro-vascular complications Assess CVD risk in context of age: impact of age of onset, age-specific goal for blood pressure and lipid profile
**Evidence**	**Best Practices**	**Future Directions**
High incidence of obesity High risk for rapid progression and poor outcomes Intensive lifestyle and medical intervention have not been efficacious Bariatric surgery is effective for weight and glycemic outcomes High socioeconomic disparities	Use of metformin + GLP-1 RA Use of intensive lifestyle management Screening for depression, social determinants of health, eating disorders Screening for complications and comorbidities	Develop efficacious lifestyle and medical/surgical interventions Use SGLT2 inhibitors^*^ Identify optimal medical treatment vs surgical interventions Identify factors to predict /stratify patients at higher risk of rapid progression
**Physical health in YA with Type 2 diabetes**		
**Evidence**	**Best Practices**	**Future Directions**
High incidence of obesity High risk for rapid progression and poor outcomes Intensive lifestyle and medical intervention have not been efficacious Bariatric surgery is effective for weight and glycemic outcomes High socioeconomic disparities	Use of metformin + GLP-1 RA Use of intensive lifestyle management Screening for depression, social determinants of health, eating disorders Screening for complications and comorbidities	Develop efficacious lifestyle and medical/surgical interventions Use SGLT2 inhibitors^*^ Identify optimal medical treatment vs surgical interventions Identify factors to predict /stratify patients at higher risk of rapid progression
**Mental Health**		
**Evidence**	**Best Practices**	**Future Directions**
***Diabetes-Related Distress*** Depressive symptoms are associated with poorer diabetes management	Clear distinction between diabetes distress and depression so that specific treatment practices can be instituted Current available tools for diabetes distress (DDS, PAID) Nonjudgmental, supportive care independent of glycemic control and outcomes	Develop an age-specific survey to assess diabetes distress and distinguish it from depression Develop interventions to improve diabetes-related distress Educate adult providers on communication/language specific to this age group
***Mental Health Disease*** Increased incidence of: Schizophrenia Bipolar disorder Depression Anxiety Depressive symptoms, which are associated with poorer diabetes management Eating disorders	Current available tools for : Depression (PHQ-2, PHQ-9, BDI) Fear of Hypoglycemia (HFS-II) Anxiety (GAD-7) Substance abuse ( CAGE-AID) Eating disorders (Diabetes Eating Problems Survey-DEPS-R) Screening tools are not consistently used in clinics due to: Lack of in-house behavioral health support Burden and consumption of time	Create a single assessment tool that could generate a standardized roadmap for identifying, screening and referring YA with diabetes and MH concerns. Assess impacts of MH screening and intervention on diabetes outcomes Assess cost-effectiveness of MH and diabetes outcomes Create an age-specific competency curriculum for clinicians and specialists to assess and support YA with MH Train more MH professionals with specific knowledge of YA and diabetes
**Quality of Life**		
**Evidence**	**Best practices**	**Future Direction**
High A1c is associated with low health-related QoL	Assessment of QoL Referrals to MH specialists with knowledge of T1D Use of T1D and Life measure (TiDAL) Ongoing screenings for MH comorbidities	Develop tools to assess QoL in YA Develop interventions to improve QoL Assess impact of interventions on QoL on diabetes outcomes and cost-effectiveness

RCT, research clinical trial; YA, young adults.

QoL, quality of life; DKA, diabetic ketoacidosis; SH, severe hypoglycemia; CVD, cardiovascular disease; YA, young adults; T1D, type 1 diabetes; ACE, angiotensin-converting-enzyme inhibitor; ARB, angiotensin receptor blocker.

^*^Awaiting study results.

GLP-1RA, Glucagon-like peptide-1 receptor agonists; SGLT-2 sodium-glucose cotransporter 2.

MH, mental health.

QoL, quality of life; MH, mental health; YA, young adults.

Next, participants focused on physical health in youth-onset T2D. YA with T2D are a growing population (32) with high rates of obesity and substantial socioeconomic disadvantage (33, 34). The clinical course, management, response to treatment, and outcomes of youth-onset T2D are different compared to adult-onset T2D. A faster loss of beta-cell function and a greater degree of insulin resistance have been described in this age group (35). The response to lifestyle modification, metformin, and insulin is poor in many cases (2). Use of a GLP1-agonist (36) and bariatric surgery (37) may be more beneficial; however there are limited long-term data. Again, transfer to adult care is associated with deterioration of glycemic control (34). Participants agreed that there is a lack of tailored guidelines and educational materials to care for persons with youth-onset T2D. Moreover, there is a need to identify best treatment options and to develop markers to identify patients with rapidly progressive T2D (2). Long-term data from the TODAY Study are forthcoming (**Table 1**).

### Mental Health

YA with diabetes are a particularly vulnerable group for mental health (MH) concerns. An increase in psychosocial issues and risk-taking behaviors, and a disruption in continuity of care have been described in this age group (38, 39). Indeed, this age group has the highest rates of MH concerns across the adult lifespan and diseases such as schizophrenia and bipolar disorders may emerge during this time (10). Notably, YA with diabetes are a greatly understudied population as they often do not participate in research. Thus, there are gaps in the literature.

At present, the MH research literature focuses mainly on depression, diabetes distress, eating disorders, anxiety, and substance abuse (40) with specific assessment tools for each concern: depression (PHQ-2, PHQ-9, BDI) (41, 42), diabetes distress (DDS, PAID) (43), eating disorders (DEPS-R) (44), Fear of Hypoglycemia (HFS-II) (45), anxiety (GAD-7) (46), and substance abuse (CAGE-AID) (47), but assessment of these multiple MH concerns can be time-consuming and burdensome for both clinicians and patients. Furthermore, there is a lack of qualified MH providers to follow-up on concerning assessments (48). The participants recommended the creation of a single assessment tool that could generate a standardized roadmap for identifying, screening, and referring YAs with diabetes for MH concerns. Moreover, they highlighted the importance of distinguishing between depression and diabetes distress in order to determine specific treatment recommendations and practices for such concerns.

Another topic discussion was how the use of language is important in all diabetes management interactions but is especially critical with this age group. Providers need to acknowledge and understand that communication and words chosen to communicate can help creating a partnership in care for this population (49). In this context, presenters highlighted the use of autonomy-supportive communication strategies such as motivational interviewing.

Intervention strategies for optimizing MH care in the YA should begin with the specific embedding of MH in overall diabetes care. It was suggested that providers meet YA where they are at and use on-demand/drop-in services, late afternoon/evening hour meetings, texting, telehealth, or other modes of communication. Other suggestions included the use of Telehealth treatment (24), enhancing YA-provider communication and problem-solving (50), structured occupational therapy (51), Peer Support/Peer Group Education (52), and a multi-component program such as LEAP (Let’s Empower and Prepare) (53). In addition, given the links between parental support and MH in childhood and adolescence (54), it was recommended that utilizing a pediatric-like family approach, including family, peers, and significant others in adult medical appointments and management decisions may permit more supportive, engaged ongoing treatment for YA with diabetes and thus may address some of the gaps in care and encourage return visits.

Furthermore, the participants agreed upon the need to develop clinical strategies that can assist YA with moving from assessment/referral to actual clinical care visits for diabetes and MH concerns. Some suggestions to aid this movement to clinical care included paying attention to and aiding patients with navigating their insurance coverage and using telehealth practices (virtual visits, virtual screenings), especially given recent reimbursement. However, many recognized the importance of ongoing Face- to-Face visits, especially in urgent MH situations. Ideally, YA with executive function deficits should receive help to navigate the health care system from care ambassadors or patient navigators, however these staff members are limited in adult care (55).

Adult providers also need to be aware that these YA might participate in risk-taking behaviors (e.g., alcohol, drugs, unprotected sex) along with their peers, who likely do not have diabetes. Suggestions for addressing these risk-taking behaviors and providing support to the YA is important; this includes maintaining a non-judgmental attitude while providing education and resources about how to manage diabetes during these situations (**Table 1**).

Finally, participants discussed the following challenges to optimizing MH treatment for YA with diabetes: limited access to care, time constraints, limited number of trained MH providers with knowledge of diabetes, long waiting lists, gaps in continuity of care, and inadequate reimbursement. Discussion also highlighted the need to assess socioeconomic status for this YA group since such deficiencies can create high risk and reduce treatment opportunities. Participants agreed that more research was needed to understand particular challenges facing YA with diabetes from lower socioeconomic backgrounds or from racial/ethnic minority groups, especially given the increased awareness of potential for care disparities (19).

### Quality of Life

Quality of life (QoL) relates to perceptions of people’s position in life in the context of culture and values in relation to their goals and expectations (56). Generally, QoL has multiple constructs (e.g., general, health-related, or diabetes-specific) and domains (physical, psychological, and/or social functioning). Any intervention aimed at preserving QoL must be explicit in its focus using appropriate, clinically-validated current measures.

Despite the availability of multiple, validated QoL measures, there are gaps, which may impact our ability to systematically and meaningfully assess QoL in YA with diabetes. First, few measures are specifically targeted to the YA (57, 58). It is possible that meaningful insights for this age group may be lost when using measures validated across the adult lifespan. QoL measures commonly face ceiling effects in which persons, despite meaningful stresses in their lives, report high QoL. The participants recommended the use of specific, validated quality of life measures, like the Type 1 Diabetes and Life (T1DAL) measures (58), which were created to ensure they remain current to modern day clinical practice and personal experience.

It has been shown that factors related to fear of diabetes-related complications can contribute to or correlate with higher or lower levels of QoL (59). The group acknowledged that routine and systematic collection of self-reported QoL and its contributing factors is a major barrier to developing clinic-based assessments and interventions to ensure preservation of QoL in this vulnerable population. The time and resources needed to capture this information are major barriers to large-scale quality improvement or clinical research efforts. There is a limited evidence base for interventions targeting QoL preservation for YA with diabetes. Not unexpectedly, there is little to no literature on the clinical efficacy and cost-effectiveness of these interventions.

Current guidelines focus on routine assessment of more narrowly-defined constructs (e.g. diabetes distress, fear of hypoglycemia, and family conflict) with appropriate referrals to knowledgeable specialists. Further, the guidelines recognize the vulnerability of this period and recommend preventative MH visits at key transition points to focus on YA issues. The limited number of trained MH professionals who work with diabetes highlights the need to provide basic training to primary care and specialty healthcare professionals to screen, provide anticipatory guidance, and potentially intervene on common factors with may adversely impact QoL (**Table 1**).

On a practical, immediately actionable level, the participants recommended assessing diabetes-related factors interfering with QoL and developing a plan to address those factors over time. This will increase the likelihood that the clinical encounter was patient-centered and impactful. Future studies are needed to develop clinic-based, scalable interventions to address critical risk factors which may jeopardize the QoL of YA with diabetes.

## Discussion

YA with diabetes face unique challenges in their management] and have a high risk of poor outcomes that can negatively impact physical health, MH, QoL and life expectancy. Thus, it is imperative for pediatric and adult clinicians to continue working in concert on targeted interventions that focus on this age group to reduce the risk of acute and long-term complications and to support and improve YA MH and QoL.

YA with diabetes have suboptimal glycemic control and are at high risk for adverse outcomes that become magnified at the time of transfer from pediatric to adult care systems. Our unique multidisciplinary participants reviewed the current literature, identified gaps, shared best practices, and highlighted needs for future research with the goal of improving the healthcare process and diabetes-related clinical outcomes for YA with diabetes.

There was substantial interest among adult providers in working with pediatric providers and MH professionals to identify best practices and to plan for future studies specific to the YA population. The results of this two-day meeting discussion are summarized in this article with the intent to be used for development of best practices and future research projects aimed at overcoming current gaps in knowledge and improving both healthcare processes and outcomes for YA with diabetes.

## Data Availability Statement

The original contributions presented in the study are included in the article/supplementary material. Further inquiries can be directed to the corresponding author.

## Author Contributions

ET, JL, RB, and LL contributed to study concept and design. ET, JL, SH, SM, and MR contributed to data acquisition, analysis, and interpretation, ET, JL, SH, SM, MR, and MM, drafted the manuscript. ET and LL critically revised the manuscript for important intellectual content. ET is the guarantor of this work and, as such, had full access to all the data in the study and takes responsibility for the integrity of the data and accuracy of the data analysis.

## Funding

We thank an anonymous philanthropic donor for this work.

## Conflict of Interest

The authors declare that the research was conducted in the absence of any commercial or financial relationships that could be construed as a potential conflict of interest.

## Publisher’s Note

All claims expressed in this article are solely those of the authors and do not necessarily represent those of their affiliated organizations, or those of the publisher, the editors and the reviewers. Any product that may be evaluated in this article, or claim that may be made by its manufacturer, is not guaranteed or endorsed by the publisher.
